# Distributions of *Xenopus* species and their helminth parasites in ecological zones of Nigeria

**DOI:** 10.1371/journal.pone.0348516

**Published:** 2026-05-19

**Authors:** Emmanuela U. Anele, Ishaya Haruna Nock, Ibrahim M. K. Gadzama, Grace S. N. Kia, Tharindu Premachandra, Joseph A. Jackson, Richard C. Tinsley, Ben J. Evans

**Affiliations:** 1 Department of Zoology, Ahmadu Bello University, Zaria, Nigeria; 2 Department of Biology, Ahmadu Bello University, Zaria, Nigeria; 3 Department of Veterinary Public Health and Preventive Medicine, Ahmadu Bello University, Zaria, Nigeria; 4 Department of Biology, McMaster University, Canada; 5 School of Science, Engineering and Environment, Salford University, United Kingdom; 6 School of Biological Sciences, Bristol University, United Kingdom; Institute of Cytology and Genetics SB RAS: FIC Institut citologii i genetiki Sibirskogo otdelenia Rossijskoj akademii nauk, RUSSIAN FEDERATION

## Abstract

African clawed frogs (*Xenopus* species) are distributed across sub-Saharan Africa, live in water, and are hosts to diverse parasites whose distributions and host-specificities are incompletely characterized. To better understand this host/parasite biodiversity, we used morphology and Sanger sequencing to characterize *Xenopus* species and their helminth parasites in several ecological zones of Nigeria. Five *Xenopus* species were identified in Nigeria (*X. fraseri*, *X. fischbergi*, *X. poweri*, *X. tropicalis*, and *X. calcaratus*), and one – *Xenopus fraseri* – was found to have a wide ecological tolerance in four different savanna ecological zones. Thirteen species of helminths from two phyla and five major lineages were isolated: camallanoid and seuratoid nematodes (roundworms), and cestode, digenean, and monogenean platyhelminths (flatworms). Based on our sample, the nematodes exhibited higher host generalism than the platyhelminths by infecting several host species and occurring in a wider breadth of ecological zones. In this study, all parasite species specialized either to a specific tissue (e.g., the bladder or pericardium) or a similar pair of tissues (e.g., esophagus and stomach or the lower intestine and rectum), which underscores the distinctiveness if different tissue ecosystems within a host. This study provides novel and molecularly confirmed insights into host and parasite species diversity, distributions, and ecological specificities in several ecological zones of Nigeria. Future efforts should focus on transition zones between ecological zones in Nigeria.

## Introduction

The co-evolutionary histories of hosts and parasites have long fascinated biologists. By studying hosts and their parasites jointly, we gain unique insights into their co-evolutionary interactions, adaptations, immune responses, and ecological sensitivities. For example, body lice co-evolved with human and non-human primate hosts, but also recently transferred hosts [1], and acquired specificity to different microhabitats on the human body [2]. The extent of congruence between host and parasite diversification can provide information to assess past geographical, evolutionary, climatic, and ecological drivers that shape faunal evolution [3–5]. In this way parasite epidemiology is relevant to understanding macroevolution. Documentation of the biological diversity and evolutionary history of parasite fauna is also important from the standpoint of biodiversity conservation, and parasite richness and diversity is often less well characterized as compared to their larger hosts.

African clawed frogs (genus *Xenopus*) are distributed across much of sub-Saharan Africa and are widely used in biomedical research and as food [6,7]. All but one *Xenopus* species is polyploid [8], and this genus contains 29 species in two subgenera: *Silurana* and *Xenopus* following the taxonomy of Evans *et al*. [8] and Furman *et al*. [9]. The chromosome number of the diploid ancestors of the subgenera *Silurana* and *Xenopus* are 20 and 18 respectively [10]. The subgenus *Silurana* comprises the only known diploid *X. tropicalis* and three tetraploid species: *X*. *epitropicalis*, *X*. *mellotropicalis*, and *X*. *calcaratus* [8]. In comparison, the subgenus *Xenopus* is represented by 25 described species, including allotetraploids, allooctoploids, and allododecaploids, and is subdivided into 3 groups: amieti, laevis, and muelleri [8]. In addition to parallel evolution of host and parasite, patterns of parasite infection are potentially influenced by allopolyploidy in *Xenopus*, wherein polyploid species are formed via the fusion of the genomes of two ancestral species. This is because allopolyploid species may acquire resistance or susceptibility to parasites that infect one or both of their lower ploidy ancestral species [11].

Along with other frogs in the family Pipidae, *Xenopus* species are primarily aquatic and live in slow moving or stagnant water as tadpoles and as adults [12]. Their aquatic lifestyle allows them to serve as definitive or intermediate hosts for several aquatic parasites [13]. The parasite fauna of *Xenopus* is extraordinarily rich with exceptional specialization to species and specific body parts, such as the cloaca, nostrils, and urinary bladder [14,15]. Within metazoan parasites, for instance, there are over 25 genera from seven invertebrate groups infecting *Xenopus*; a richer assemblage than in most other anurans [15,16]. This diversity reflects a dual origin of the parasites, which are derived from amphibian-specialist groups but also from fish-specialist groups that likely transferred to *Xenopus* due to overlap in habitat and diet with fish [15]. Among the parasites of *Xenopus*, helminths (principally nematodes and members of the major platyhelminth lineages, Monogenea, Digenea and Cestoda) are distinguished by morphology, the site of infection within the host, and the host species. *Xenopus* species have a complex web of interactions with other organisms [15] and may serve as intermediate hosts for helminth infections of aquatic predators. Some of the nematodes occurring in *Xenopus* have direct life cycles, whilst others (camallanids and cephalochlamydid cestodes) utilize copepods as intermediate hosts. Digeneans have two or more intermediate hosts, with a mollusk as the first intermediate host. There are no known intermediate hosts for monogeneans; they are transmitted by a swimming infective stage [15].

Being amphibians, *Xenopus* belongs to the most threatened vertebrate class, with over 40% of species in decline [17]. Infectious diseases are a major contributor to the decline of amphibian species [18] and the diversity and conservation status of anuran parasites is poorly characterized. A better understanding of host/parasite relationships in *Xenopus* is thus relevant to biodiversity conservation.

Nigeria is situated in West Africa and is home to an extraordinary diversity of species including components of the Guinean Forest biodiversity hotspots [19], and several other diverse ecoregions [20]. Nigeria is the most populous country in Africa (~240 million people as of 2025), whose population size is forecasted to increase at a much higher rate than the global average over this century [21]. Consequently, study of Nigerian fauna is pressing from the standpoint of understanding the influences of humans (e.g., climate change, habitat alteration and loss, pollution). In this study, we present results from seven of the nine ecological zones in Nigeria: the Sahel, Sudan, Guinea, and Derived Savanna, the Jos Plateau, Montane, and Lowland Rainforest. The four types of savanna zones are distinguished by several factors including temperature, rainfall, and anthropogenic activity, and the vegetation varies from tall grasses and sparse trees (Guinea Savanna) to sparse grass and thorny bushes (Sudan Savanna). Compared to the savanna ecological zones, the montane ecological zone is characterized by lower temperatures, higher rainfall, and a high diversity of endemic fauna. The Jos Plateau ecological zone is a mosaic of montane woodland, scrublands and grasslands. The Lowland Rainforest ecological zone is characterized by tall dense and diverse trees.

Previous studies of *Xenopus* species and their parasites in Nigeria have focused on morphology [22,23]. This study aims to build on these studies by using molecular data to characterize distributions of *Xenopus* hosts and their parasites in Nigeria. We further aim to evaluate the null hypotheses of strict host/parasite co-evolution in this incompletely studied system, with the predictions that parasite species have species-specific infections of hosts, and that phylogenetic relationships among the hosts and their parasites are congruent. We additionally aimed to qualitatively characterize the extent of habitat specificity of parasites of Nigerian *Xenopus* in terms of the number of host species and tissue types they infect, and the number of distinct ecoregions in which they occur.

## Results

### Distributions of *Xenopus* species in Nigeria

**Fig 1:**
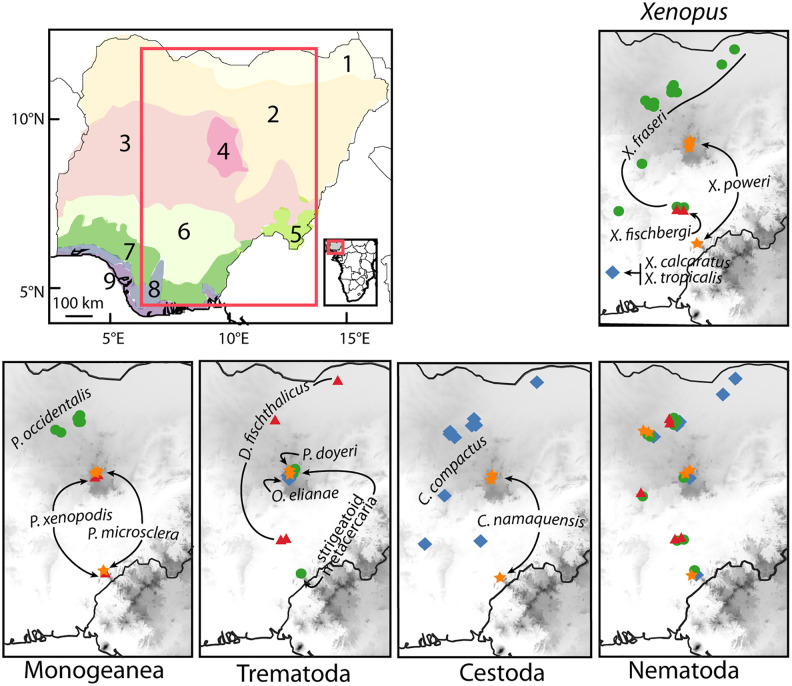
Distribution of ecological zones in Nigeria following [20] (top left), host *Xenopus* species (top right), and four major groups of *Xenopus* parasites (bottom row). Numbered ecological zones are: (1) Sahel Savanna, (2) Sudan Savanna, (3) Guinea Savanna, (4) Jos Plateau, (5) Montane Forest, (6) Derived Savanna, (7) Lowland Rainforest, (8) Freshwater Swamp Forest, and (9) Coastal Vegetation. A small red box on the inset in the top left panel shows the location of Nigeria; the large red box on this panel demarcates the area depicted in other panels, which illustrate sampling localities of frogs (top right) and parasites (bottom row). Parasite species are labeled except the nematodes where species are represented by the following symbols: *Camallanus kaapstaadi*: blue diamonds; *Chabaudus leberrei*: green circles; *Batrachocamallanus occidentalis*: red triangles; *Batrachocamallanus xenopodis*: orange star.

A total of 336 *Xenopus* individuals from five species were collected across seven ecological zones of Nigeria (Fig 1, S1 Table). All of the frogs we collected were adults. *Xenopus fraseri* (225/336 individuals; 67% of the collection) was the most abundant and widely distributed, and was collected in the Sahel, Sudan, Guinea and Derived Savanna ecological zones. *Xenopus fischbergi* (7/336 individuals; 2%) was the least abundant and was collected from two locations within the Derived Savanna ecological zone. *Xenopus tropicalis* and *X. calcaratus* (14/336; 4%) were collected from one locality in the Lowland Forest ecological zone. We sequenced seven of 14 samples; all of these had *X. tropicalis* mitochondrial sequences, but this sample included at least one admixed individual (EUA0334) described elsewhere [24], and for this reason we are unable to conclusively assign specimens from this locality to *X. tropicalis* or *X. calcaratus*. *Xenopus poweri* (90/336 individuals; 27%) was collected in the Jos Plateau and Montane Forest ecological zones. Slightly more females were collected than males (across all species, 183 females, 153 males; S2 Table). We did not observe a significant difference between the sexes in the mean number of parasites per individual (the mean of the female-male difference in abundance across all parasite species in all hosts:–0.35, 95% confidence interval of this mean difference: –3.10–1.29).

### Distributions and host specificities of *Xenopus* parasites in Nigeria

In total, thirteen species of helminths from two phyla and five major lineages were isolated: camallanoid and seuratoid nematodes (roundworms), and cestode, digenean, and monogenean platyhelminths (flatworms). These were present in three host species collected from seven ecological zones (Fig 1, Table 1, S3 Table).

**Table 1:** Observed distributions of parasites in *Xenopus* host species, Nigeria ecological zones, and *Xenopus* host organs.

Parasite Phylum/Class	Parasite species	Host species	Ecological zones	Host organs
Nematodes: Camallanidae	*Camallanus kaapstaadi*	a,b	a,b,c,d,e,f	esophagus, stomach
	*Batrachocamallanus xenopodis*	a,b	a,b,c,d,e,f	esophagus, stomach
	*Batrachocamallanus occidentalis*	a,c	a,b,c,d,e,f	esophagus, stomach
Nematodes: Seuratoidea	*Chabaudus leberrei*	a,b,c	b,c,d,e,f	lower intestine, rectum
Platyhelminthes: Cestoda	*Cephalochlamys compactus*	a,c	a,b,c,d	intestine
	*Cephalochlamys namaquensis*	a	e,f	intestine
Platyhelminthes: Trematoda (Digenea)	*Diplodiscus fischthalicus*	b	a,b,d,	rectum
	*Progonimodiscus doyeri*	a	e	rectum
	*Oligolecithus elianae*	a	e	intestine
	strigeatoid metacercaria*	a	e,f	pericardium
Playhelminthes: Monogenea	*Protopolystoma occidentalis*	b	b,c	urinary bladder
	*Protopolystoma xenopodis*	a	e,f	urinary bladder
	*Protopolystoma microsclera*	a	e,f	urinary bladder

Ecological Zones: a: Sahel Savanna; b: Sudan Savanna; c: Guinea Savanna; d: Derived Savanna; e: Jos Plateau; f: Montane

Host species: a: *X. poweri*; b: *X. fraseri*; c: *X. fischbergi*

* this is a developmental stage of digenean parasites that could not be identified to species.

**Fig 2:**
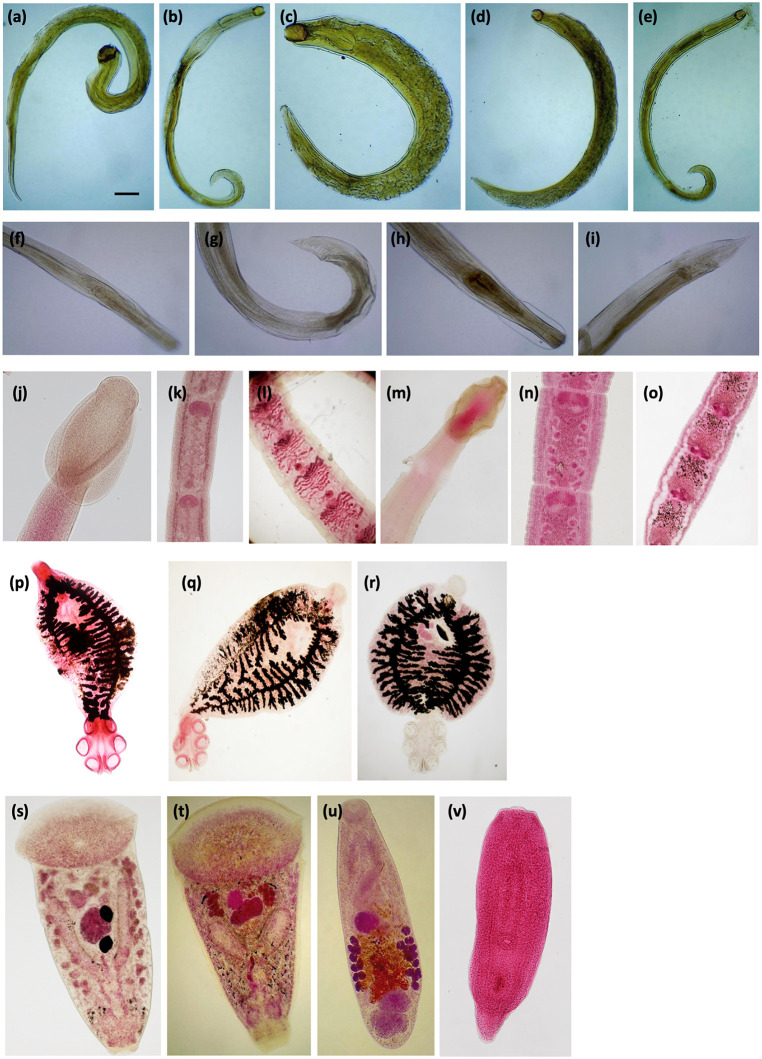
Photographs of (a) a female *Camallanus kaapstaadi*; (b) a male *Batrachocamallanus sp.*; (c) a female *Batrachocamallanus xenopodis*; (d, e) a female and male *Batrachocamallanus occidentalis*, respectively; (f,g,h,i) anterior & posterior views of a male and female*Chabaudus leberrei,* respectively; (j,k,l) scolex, immature proglottid, and gravid proglottid of *Cephalochlamys compactus*,, respectively; (m,n,o) scolex, immature proglottid, and gravid proglottid of *Cephalochlamys namaquensis*, respectively; (p) *Protopolystoma xenopodis*; (q) *Protopolystoma microsclera*; (r) *Protopolystoma occidentalis*; (s) *Diplodiscus fischthalicus*; (t) *Progonimodiscus doyeri*; (u) *Oligolecithus elianae*; (v) Strigeatoid metacercaria (a larvae of an unidentified digenetic trematode). The scale bar of 5 mm for all panels is in (a).

In our sample, nematodes were the most abundant and widely distributed helminth and were isolated from three host species within six ecological zones. Camallanoid nematodes sampled included: (i) *Camallanus kaapstaadi* (Fig 2a) which was isolated from the esophagus and stomach of *X. fraseri* and *X. poweri*, (ii) *Batrachocamallanus xenopodis* (Fig 2c) which was isolated from the esophagus and stomach of *X. fraseri* and *X. poweri*, and (iii) *Batrachocamallanus* *occidentalis* (Fig 2d) which was isolated from the esophagus and stomach of *X. fraseri* and *X. fischbergi*. Thus, the relatively distantly related host species *X. fraseri* and *X. poweri* were both host to the camallanoid nematodes *C. kaapstaadi* and *B. xenopodis*. These two species plus *X. fischbergi* were all host to the another morphologically-identified seuratoid nematode species *Chabaudus leberrei* (Fig 2e), even though these host species occupy distinctive ecological zones: the savannas – Sahel, Sudan, Guinea, and Derived – and montane grassland of the Jos Plateau, respectively (though *C. leberrei* was not detected in the Sahel).

**Fig 3:**
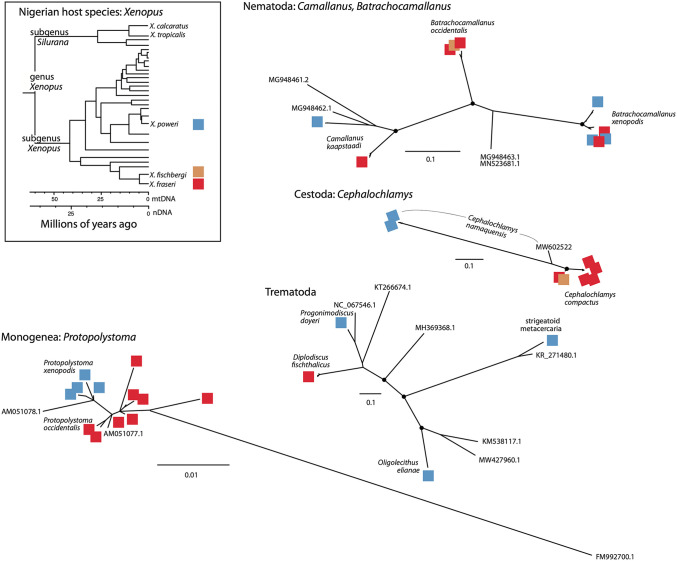
Phylogenetic inferences among Sanger sequences of Nigerian *Xenopus* species (top left insert) and their helminth parasite (Nematoda, Platyhelminthes: Cestoda, Trematoda (Digenea) Monogenea); black circles over nodes indicate bootstrap support of at least 90%, except for terminal nodes where this is omitted for clarity. For the host phylogeny, two divergence time estimates are provided based on mitochondrial (mtDNA) and nuclear DNA (nDNA) data, as described in [8].

Two species of platyhelminth cestode (genus *Cephalochlamys*) were isolated. *Cephalochlamys namaquensis* (Fig 2h) and *C. compactus* (Fig 2f) were isolated from intestines of *X. poweri* and from *X. fraseri* + *X. fischbergi*, respectively. That *X. fraseri* and *X. fischbergi* were host to the same species of cestode (*C. compactus*) and nematode (*B. occidentalis*; see above), is consistent with their close phylogenetic affinities, and their co-occurrence in the Derived Savanna ecological zone.

In platyhelminth parasites, several other examples of host-specificity were detected in our sample. For instance, the trematode parasites *Progonimodiscus doyeri* (Fig 2m) and *Oligolecithus elianae* (Fig 2n), the monogenean parasites *Protopolysoma xenopodis* (Fig 2i) and *P. microsclera* (Fig 2j), and an unidentified digenean parasite at the strigeatoid metacercariae developmental stage (Fig 2o) were detected only in *X. poweri*. Likewise, the monogenean parasite *Protopolysoma occidentalis* (Fig 2k) and the trematode parasite *Diplodiscus fishthalicus* (Fig 2l) were detected only in *X. fraseri*. But it remains unclear whether these parasites are host-specific or whether this is a consequence of incomplete sampling.

Phylogenetic relationships inferred among Sanger sequences of *Xenopus* host species and their helminth parasites are presented in Fig 3. As discussed above, nematodes (camallanoids and seuratoids) exhibited host generalism by infecting several host species (e.g., *C. kaapstaadi* and *B. occidentalis* infect *X. fraseri* and *X. poweri*, *C. leberrei* infects *X. fraseri, X. fischbergi* and *X. poweri*). In contrast (and based on our sample), platyhelminthes (cestodes, digeneans, monogeneans) tended to exhibit higher host specificity (e.g., in our sample *C. namaquensis* was found only in *X. poweri*, *O. elianae and P. doyeri* were found only in *X. poweri*, *P. occidentalis* was found only in *X. fraseri*).

There were also examples where phylogenetically diverged parasite species co-infected the same host species. For example, species from at least three different trematode genera and at least two different nematode genera can infect *X. poweri* (Fig 3). Similarly, at least three diverged species of nematode in two genera can infect *X. fraseri* (Fig 3). Interestingly, however, the diversity of monogenean parasites in the genus *Protopolystoma* was comparatively modest with different but only modestly diverged species infecting *X. fraseri* and *X. poweri* (Fig 3). Attempts to amplify the DNA sequences of the monogenean *P. microsclera* and the nematodes *C. leberrei* and *Batrachocamallanus* sp. were unsuccessful, and these taxa thus were not included in the molecular analyses.

Higher niche generalism of nematodes as compared to platyhelminths is also evidenced by differences in diversity of ecological zones they occupy (S3 Table). Nematode species were sampled in five or six ecological zones, whereas platyhelminth species were detected in only one, two, or three ecological zones.

All parasite species detected in this study infected only one tissue, with the exception of three nematode species that infected the esophagus and stomach and one that infected the lower intestine and rectum (Table 1). Both of these pairs of tissues are closely associated portions of the digestive tract, though differences do exist, for example in acidity and rugosity of the lining. Overall these observations highlight the distinctive ecological conditions of different tissue types – and the commensurate specialization of the parasites that infect them.

## Discussion

This study is currently the most comprehensive, molecularly confirmed perspective on the distributions of *Xenopus* species and their helminth parasites in Nigeria. *Xenopus fischbergi* is a recently described species [8] and the taxonomic status of *X. fraseri* was recently clarified [25]; both of these species formerly were referred to as *X. muelleri* (e.g., Kobel *et al*. 1996). It is therefore unclear whether earlier reports of the earlier use of species names “*X. muelleri*” in the forest ecological zones of Nigeria [26] and “*X. fischbergi*” in the Guinea Savanna ecozone of Nigeria [22] actually are *X. fraseri* or *X. fischbergi* because molecular tools were not employed for these earlier species identifications. This study provides additional information on the distribution of *X. fraseri* in the Sahel savanna and three other vegetation zones, and expands its geographical range to include Nigeria, which is perhaps unsurprising because Nigeria lies between previously known localities on either side of Nigeria (in northern Ghana and Cameroon) [25]. The high abundance of *Xenopus fraseri* in four savanna ecological zones of Nigeria (Sahel, Sudan, Guinea and Derived Savanna) indicates that this frog species has a wide ecological tolerance. *Xenopus fraseri* was collected in the Derived Savanna zone in the same location as *X. fischbergi*, its close relative*.* Derived Savanna forms as a result of intense anthropogenic activities on the Lowland Forest ecological zone [20], which suggests *X. fischbergi* also tolerates disturbed habitats. The holotype of *Xenopus fischbergi* was collected on the Jos Plateau in Nigeria, even though surveys in this study only detected *X. poweri* in this locality. *Xenopus fischbergi* also has a wide range in western Africa [8,25]. *Xenopus poweri* was previously identified as a subspecies of *X. laevis* (*Xenopus laevis sudanensis*) [27], or within *X. laevis sensu lato* [9]. The presence of *X. poweri* on the Jos Plateau and Montane Forest Ecozone (both >1200 m) [20] suggests that this species occurs in areas with high altitude in multiple ecological zones.

*Xenopus tropicalis* and *X. calcaratus* were collected from the Lowland Forest ecological zone. Several studies documented *X. tropicalis* in forest habitat [26,28], which is consistent with our findings, but this could also refer to *X. calcaratus*. Anele *et al*. [22] collected *X. tropicalis* from a relatively undisturbed location within the northern Guinea Savanna ecological zone, indicating occupancy of this species in both savanna and forest ecozones.

Generalist species are those with broad ecological tolerances and/or diets whereas specialist species have more narrow ecological niches, dietary needs, or other requirements [29]. It is sometimes assumed that specialists do better in their optimal habitat whereas generalists often do better in variable habitats [30]. One way to characterize parasite ecology is to define species that infect multiple distantly related species as host generalists and those that infect one (or few) closely related species as host specialists. According to these definitions – and based on our sample – the generalists include nematodes - *C. kaapstaadi*, *B. xenopodis* and *C. leberrei* and the specialists include all of the trematodes, cestodes and monogeneans isolated in this study. The camallanid nematode *B. occidentalis* has intermediate host specificity in that it infects two closely related species (*X. fraseri, X. fischbergi*).

Another approach would be to classify parasites based on the number of ecological zones they were found in (e.g., specialists occupy one whereas generalists occupy more than one), irrespective of their host specificity. Under this approach the generalists include all nematodes, cestodes, monogeneans and trematodes (digeneans), except *P. doyeri* and *O. elianae* which are specialists to the Jos Plateau. More realistically, niche specialization could be considered a continuous trait with cestodes, monogeneans and trematodes being more specialized because they occupy only 1–3 ecological zones and nematodes being more generalized because they occupy 5–6.

Specimens of *X. tropicalis* and *X. calcaratus* (both are in subgenus *Silurana*) that were already preserved in ethanol were examined for helminths, but none were detected. It is possible that this is a consequence of the small sample size (14 individuals) or because these specimens were handled differently from the others. Other surveys have recorded infections in subgenus *Silurana* of several parasite species that also infect the sister subgenus *Xenopus* [28]. In general, species in subgenus *Silurana* tend to have low abundance and diversity of helminths (JAJ and RCT, personal observation).

Nematodes had the highest overall prevalence in the *Xenopus* species we examined. The prevalences of members of the genera *Camallanus* and *Batrachocamallanus* in this study are summarized in Supplemental Table 2, and have been reported from other *Xenopus* species and places as well [22,23]. *Batrachocamallanus occidentalis* and *B. xenopodis* are known to infect *X. muelleri*, which is distributed in East Africa [31]. Hence their presence in *X. fraseri* and *X. fischbergi* – both close relatives of *X. muelleri* – was not unexpected. However, *X. fraseri* seems to harbour several of the same parasites as *X. poweri*, indicating some degree of host generalism for *B. xenopodis* and *C. kaapstaadi*. *Camallanus kaapstaadi* has a wide distribution in sub-Saharan Africa and infects *X. laevis* other species in the laevis group, as well as *X. fraseri* [31]. The presence of the nematode *B. occidentalis* in the esophagus and stomach of *X. fischbergi* and *X. fraseri* but not *X. poweri* could indicate a degree of host specificity. The nematode *Chabaudus leberrei* was isolated from the lower intestine and rectum of *X. fraseri, X. fischbergi,* and *X. poweri*, which indicates that this parasite is a *Xenopus* generalist. Consistent with this, *C. leberrei* is also known to infect other species in the muelleri and laevis groups [32]. *Cephalochlamys compactus* was detected in the closely related host species *X. fraseri* and *X. fischbergi*, whereas *C. namaquensis* showed host specificity for *X. poweri*. *Cephalochlamys namaquensis* is also known to infect the laevis group, and *C. compactus* occurs in *X. muelleri* in addition to *X. fischbergi* and *X. fraseri* [33]. The presence of *Oligolecithus elianae* in the intestine of *X. poweri* from the Jos Plateau but not in *X. poweri* collected from the Montane ecozone could indicate habitat specificity or alternatively incomplete sampling. Previous studies have recorded this parasite from *X. l. laevis* and *X. l. victorianus* from diverse areas in Africa (the Democratic Republic of the Congo, South Africa, Uganda, Rwanda) [34].

The presence of the digeneans *P. doyeri* and *D. fischthalicus* in the rectum of *X. poweri* and *X. fraseri* respectively could be facilitated by sympatry of these host species. This could indicate a degree of generalism for both parasites. *Progonimodiscus doyeri* is known to infect species in the *Xenopus* subgenus *Xenopus*, including hosts in the laevis and muelleri groups [11], but it has not been found in the subgenus *Silurana*. *Diplodiscus fischthalicus* is known to infect the rectum of *X. poweri* and has been isolated from the rectum of *Hoplobatrachus occipitalis*, which is a ranid frog [22]. Using morphology, three species of *Protopolystoma* were identified from the urinary bladder of *Xenopus* species. These were confirmed with DNA isolated from *P. xenopodis* and *P. occidentalis,* though DNA extraction was not achieved from *P. microsclera*. The occurrence of *P*. *xenopodis* and *P. microsclera* in a single *X. poweri* individual is unusual, as is the high abundance of *Protopolystoma* per individual host (as high as 7 and 9 parasites in *X. fraseri* and *X. poweri*, respectively). More typically, only one *Protopolystoma* individual is found per host individual [3,35,36]. In our sample, *Protopolystoma* species are highly host specific, with *P. xenopodis* and *P. microsclera* infecting *X. poweri* and *P*. *occidentalis* restricted to *X. fraseri*.

In this study, host specificity was observed in cestodes (*C. namaquensis*), digeneans (*O. elianae*) and monogeneans (*P. xenopodis* and *P. microsclera*) infecting *X. poweri*, as well as *P. occidentalis* infecting *X. fraseri.* However, nematodes showed a wider range of host selection, with *B. xenopodis* and *C. kaapstadi* infecting both *X. fraseri* and *X. poweri* and *C. leberrei* infecting *X. fraseri, X. fischbergi and X. poweri*. However, one nematode species (*B. occidentalis*) and one cestode species (*C. compactus*) were found to infect only the closely related host species *X. fraseri* and *X. fischbergi*.

In several cases, we observed infection of multiple, physically connected tissue types (*C. kaapstaadi, B. xenopodis* and *B. occidentalis* from the esophagus and stomach of three different *Xenopus* species; *Chabaudus leberrei* from the lower intestine and rectum of three different *Xenopus* species). Food regurgitation in *Xenopus* [37,38] may facilitate exchange of parasites between the stomach and esophagus.

There are several caveats to our conclusions. For instance, sampling of parasites was performed over two years, and seasonal variation could have differently influenced parasite abundance. We also note that a relatively small sample of *X. fischbergi* was available for analysis and we were unable to isolate parasites from *X. tropicalis* or *X. calcaratus* (possibly as a consequence of preservation conditions); clearly a larger sample would provide a more comprehensive perspective on parasite abundance and specificity in these host species and across the ecological zones that they inhabit. Additionally, the sizes and developmental stages of some parasites hampered efforts to infer taxonomy based on morphology or DNA sequences; this limited our ability use these specimens to inform our understanding of parasite distributions and ecological tolerances. As well, our sampling efforts were restricted to Nigeria and patterns observed in this country may not be broadly representative – particularly in widespread species. Moreover, *Cephalochlamys* species, *C. leberrei*, and *D. fischthalicus* can occur in non-*Xenopus* hosts [15], so at some level are quite generalistic.

## Conclusions

African clawed frogs (*Xenopus*) and their parasites are a complex and fascinating system with which to explore host-parasite relationships, specialization to different habitat types, tissues, and host species, and to better understand biodiversity. In this study we have explored diversity and ecological specialization of this system with a focus on representatives from Nigeria. Our analyses increase understanding of the distributions of *Xenopus* and several parasites in Nigeria, identify several examples of host specialization where parasites occupy only one host species (*O. elianae*, *P. doyeri*, *C. namaquensis*, in *X. poweri* and, *D. fischthalicus* in *X. fraseri*), one habitat type but several host species (*C. compactus* from *X. fraseri* and *X. fischbergi* within the Derived Savanna), and one tissue type but several species or habitat types (*C. leberrei* in the intestine of *X. fraseri, X. fischbergi* and *X. poweri* in all the sampled ecological zones except the Sahel Savanna and the Lowland Forest ecozone). As well, we identified several instances of host generalism, where parasites infect several habitat types (all the helminths isolated in this study except *P. doyeri* which was present in the Jos Plateau only), or multiple distantly related *Xenopus* species (*C. leberrei, C. kaapstaadi* and *B. xenopodis* from *X. fraseri* and *X. poweri*). Our efforts are accompanied by voucher specimens, genetic samples, and Sanger sequences that are publicly available at a museum collection (Museum of Comparative Zoology) and a database (GenBank) that will assist with future studies of this dynamic system.

## Materials and methods

### Collections

This research was approved by the Committee on Animal Use and Care of Ahmadu Bello University (Approval #: ABUCAUC/2020/Zoology/022) and the Animal Use Committee of McMaster University (AUP #: 17-12-43). Export of specimens and samples from Nigeria was authorized by the Federal Ministry of Agriculture and Rural Development Department of Veterinary and Pest Control Services (VSD/269/S.2/3202). Euthanasia was performed via transdermal overdose of Ethyl 3-aminobenzoate methanesulfonate (Sigma).

African clawed frogs (*Xenopus* species) were collected from twenty-two locations using baited funnel entrance traps within seven ecological zones of Nigeria: Sudan Savanna, Sahel Savanna, Guinea Savanna, Derived Savanna, Jos Plateau, Lowland Forest, and Montane Forest [S4 Table; 20]. Collections of *X. tropicalis* were in 2022; all other specimens (parasites and frogs) were collected in 2020 or 2021 (S4 Table). For twenty-one of these localities, live *Xenopus* were transported from each sampled location except the Lowland Forest locality to the Entomology and Parasitology Laboratory in the Department of Zoology, Ahmadu Bello University for parasitological study. For the Lowland Forest locality only, animals were euthanized prior to transport. Because collection efforts varied among sites, we also do not attempt to compare abundances across ecological zones.

Each individual frog was euthanized and dissected; sex was determined following dissection based on visual identification of ovaries or testes. A sample of liver was excised and preserved in 100% ethanol for DNA extraction. Within 72 hours of collection, the skin and internal organs (gastrointestinal tract, heart, lungs, urinary bladder and buccal cavity) were examined for parasites using a dissecting microscope, following methods described previously [28,39,40]. The excised organs were placed separately in petri dishes containing 0.7% NaCl saline and examined for parasites. Isolated helminth parasites were relaxed in hot (not boiling) water and preserved in 70% and 100% ethanol for morphological identification and molecular analyses, respectively.

Helminths preserved in 70% ethanol were prepared for microscopy using methods described by [28,32,33,40]. Nematodes (round worms) were cleared with glycerol and viewed with a light microscope. Platyhelminthes (flatworms) including cestodes, digeneans and monogeneans were stained with aceto-carmine, dehydrated in graded series of ethanol, cleared with xylene and mounted on a glass slide with Canada balsam. Prepared slides of the flatworms were viewed with an inverted microscope (bright field imaging). Helminths were identified following [31–34,41–43]. Voucher specimens of preserved *Xenopus*, genetic samples of these frogs (liver in ethanol), and parasites are archived in the Harvard Museum of Comparative Zoology (accession numbers: MCZ IZ 168202–168895; S5 Table).

A bootstrap approach was used to evaluate the null hypothesis of no sex difference in parasites abundance. As a test statistic, we used the mean of the differences between the mean number of parasites detected in females and males across all species and parasites. We then generated a distribution of statistics by resampling the observed differences with replacement. A 95% confidence interval was estimated as the first and last 2.5^th^ percentile of this distribution.

### Molecular data

DNA was extracted from liver tissues and whole bodies of helminths of representative specimens from each collection locality using the DNEasy extraction kit (Qiagen) following the manufacturer’s protocol. For frogs, species identification was achieved by Sanger sequencing of a portion of the 16S rDNA of *Xenopus* that was amplified using 16SC_L and 16S_D primers [44]. For the nematodes, the COI was amplified and sequenced using LCO1490/ HC02198 and previously reported thermocycling conditions [45,46]. For cestodes and trematode parasites, a portion of the COI gene was amplified and sequenced using either Dice 1F/Dice 14R or Dice 1F/Dice 11R primers and using previously reported thermocycling conditions [S6 Table; [47]]. For monogenean parasites, a portion of the 18S rRNA was amplified using the primer pair F18/ IR5 and previously reported thermocycling conditions [48]. Amplified 18S rRNA was then sequenced with a combination of 18F1, 18F2, 18F3, 18RA, 18RB, 18RC, 18RG sequencing primers as detailed in S6 Table. Forward and reverse sequences were merged into a consensus using Geneious software version 2023.1.1 (Dotmatics) and aligned manually using Mesquite version 1.12 [49].

New data from Nigerian fauna were combined with data from public archives (GenBank accession numbers: MG948463.1, MN523681.1, MG948462.1, MG948461.1, KM538117.1, MW427960.1_6885–7480, NC_067546.1_7100–7532, KT266674.1_6952–7560, MH369368.1, KR271480.1, MW602522, AM051078.1, AM051077.1, FM992700.1, AP014695). Phylogenetic relationships among sequences within *Xenopus* and within each helminth group were estimated using maximum likelihood as an optimality criterion and a model of evolution that was selected using the Bayesian Information Criterion using IQ-TREE version 2.3.6 [50]. The following models of evolution were selected for each dataset: *Xenopus*: GTR + F + R3, cestodes: HKY + F + I, trematodes: TIM + F + I + G4, nematodes: TN + F + G4, and monogeneans: K2P, with model acronyms defined in the IQTree documentation (http://www.iqtree.org/doc/Substitution-Models).

## Supporting information

S1 Table Number of *Xenopus* specimens collected in different ecological zones of Nigeria by species. (XLSX)

S2 Table Number of infected host individuals and mean number of parasite individuals, including all parasite species, per infected individual by host sex. Numbers in parentheses are standard errors of the mean.
(XLSX)

S3 Table Number of parasite individuals detected (count), mean number of parasites per infected individual (intensity) by host species (Host), tissue of infection (Tissue), and ecological zone. Numbers in parentheses are standard errors of the mean.
(XLSX)

S4 Table Information on sample localities by ecological zone. (XLSX)

S5 Table Information on Xenopus samples examined in this study including the Museum identification number (Museum ID), each of two field identification numbers (Field ID, Other ID), the species (Species), Sex (Sex), Locality, and whether mitochondrial DNA was sequenced (MtDNA Seq). (XLSX)

S6 Table PCR and sequencing primers used in this study. (XLSX)
